# Study on the Approach to Obtaining Mechanical Properties Using Digital Image Correlation Technology

**DOI:** 10.3390/ma18081875

**Published:** 2025-04-19

**Authors:** Shuai Wang, Bin Wang, Shengyong Mu, Jianlong Zhang, Yubiao Zhang, Xiaoyan Gong

**Affiliations:** 1 College of Mechanical Engineering, Xi’an Shiyou University, Xi’an 710065, China; shuai.wang.ac@outlook.com; 2 Department of Mechanical and Aerospace Engineering, Brunel University of London, Uxbridge UB8 3PH, UK; 3 School of Mechanical Engineering, Xi’an University of Science and Technology, Xi’an 710054, China; zhangyubiao@stu.xust.edu.cn (Y.Z.); gongxymail@163.com (X.G.); 4 R&D Department, Xi’an Special Equipment Inspection Institute, Xi’an 710065, China; mushengyong@xseii.com.cn (S.M.); zhangjl@chd.edu.cn (J.Z.)

**Keywords:** uniaxial tensile test, elastoplastic finite element method, mechanical properties

## Abstract

Accurate mechanical property parameters constitute an indispensable guarantee for the accuracy of finite element simulations. Traditionally, uniaxial tensile tests are instrumental in acquiring the stress–strain data of materials during elongation, thereby facilitating the determination of the materials’ mechanical property parameters. By capitalizing on the digital image correlation (DIC) non-contact optical measurement technique, the entire test can be comprehensively documented using high-speed cameras. Subsequently, through in-depth analysis and meticulous numerical computations enabled by computer vision technology, the complete strain evolution of the specimen throughout the test can be precisely obtained. In this study, a comparison was made between the application of strain gauges and DIC testing systems for measuring the strain alterations during the tensile testing of 316L stainless steel, which serves as the material for the primary circuit pipelines of pressurized water reactor (PWR) nuclear power plants (NPPs). The data procured from these two methods were utilized as material mechanical parameters for finite element simulations, and a numerical simulation of the uniaxial tensile test was executed. The results reveal that, within the measuring range of the strain gauge, the DIC method generates measurement outcomes that are virtually identical to those obtained by strain gauges. Given its wider measurement range, the DIC method can be effectively adopted in the process of obtaining material mechanical parameters for finite element simulations.

## 1. Introduction

The finite element method plays a vital role in engineering design and simulation. Precise material mechanical properties are among the key factors that ensure the accuracy of finite element simulation results. Material mechanical parameters are commonly determined using uniaxial tensile tests, with accurate measurements of stress–strain data being paramount. When it comes to strain measurement, traditional methods like extensometers and strain gauges have their drawbacks, including a limited measuring range, significant installation errors of measuring tools, and an inability to capture the features of material necking [1,2]. The digital image correlation method is a non-contact, high-precision measurement technique that employs computer vision technology for image processing and numerical calculations to obtain full-field deformation and strain fields at different scales [3,4]. This approach can be applied to measurement areas ranging from very small to large, and its results are easily comparable to finite element results or strain gauge measurements. The DIC method, a technique first proposed in the 1980s, is widely used in laser speckle metrology for surface displacement measurement and its use has witnessed substantial growth, thanks to the pioneering efforts of scholars and researchers in the field. This progress encompasses various dimensions, including the selection of functions, the enhancement of speckle quality, and the development of interpolation algorithms. These advancements have led to significant improvements in DIC’s computational accuracy and speed in measuring displacements [5,6,7,8]. In recent years, the focus of DIC research has shifted primarily towards broadening its range of applications. Researchers have employed DIC to measure the Young’s modulus of aluminum alloys, validating its precision through comparisons with strain gauge measurements [9,10]. By harnessing DIC’s full-field measurement capabilities, scholars have explored the impact of factors such as specimen size on the Young’s modulus. Additionally, DIC has proven valuable in assessing Poisson’s ratio, anisotropic plastic ratio parameters, and flow curves. It has also found applications in high-temperature tensile experiments [11]. Behrad successfully employed experimental DIC methods to predict the stress–strain response of 304 stainless steel specimens across a temperature range spanning from room temperature to 900 °C [12]. Shen et al. combined finite element analysis with DIC measurements to investigate the uniaxial tensile properties of aerospace composite materials at elevated temperatures, highlighting the consistency between finite element predictions and DIC experiments [13]. In addition to tensile mechanical properties, digital image correlation (DIC), combined with complementary testing techniques such as acoustic emission (AE), X-ray computed tomography (X-CT), and scanning electron microscopy (SEM), proves invaluable in scrutinizing residual stresses, damage progression, and crack propagation [14,15,16]. Zhu et al. utilized miniature ring-core cutting alongside DIC techniques to ascertain residual interfacial stresses in thermal barrier coatings [17]. Zhang et al. integrated DIC with scanning electron microscopy to analyze the deformation and fracture behaviors of pure titanium under various loading conditions, including uniaxial, notched tension, and shear loading [18]. DIC, extending beyond its non-contact, non-destructive, and full-field measurement capacities, facilitates localized displacement measurements and provides in situ monitoring capabilities. The technique has proven highly effective in characterizing the local mechanical properties of non-uniform materials like welded joints and in crack detection [19,20,21]. In summary, DIC surpasses traditional strain measurement methods, offering clear advantages. The experimental data derived through DIC plays a pivotal role in supporting advancements in material damage assessment, fracture mechanics, and finite element methods. Recent studies by Oka et al. and Novich et al. highlight DIC’s potential for nuclear-grade steels [22,23], but systematic validation for 316L under reactor-relevant conditions remains lacking.

In this study, numerical analyses and experiments were carried out to investigate the process of strain variation during uniaxial tensile tests, and the results can help guide the accurate acquisition of mechanical property parameters (curves) required during finite element simulation.

## 2. Experiment and Procedure

To accurately capture the strain variations during uniaxial tensile testing of materials, this study employed two distinct methods: strain gauges and digital image correlation (DIC) techniques. The strain distribution was assessed using these methods, while a uniaxial tensile testing machine was utilized to apply the loading load. 316L nuclear-grade austenitic stainless steel has been used as the material of the primary circuit pipeline of pressurized water reactor (PWR) nuclear power plants (NPPs) due to its strength and good corrosion resistance [24]. Within the framework of uniaxial tensile experiments, the assessment of material strain was carried out by means of both strain gauge measurements and DIC techniques. For each of these methodologies, three independent experimental trials were meticulously conducted. This comprehensive experimental approach ultimately led to the generation of a cumulative total of six datasets. The stress–strain changes experienced by 316L austenitic stainless steel during the uniaxial tensile experiment were analyzed by using numerical simulation methods and compared with the experimental results obtained.

### 2.1. Specimen

**Figure 1:**
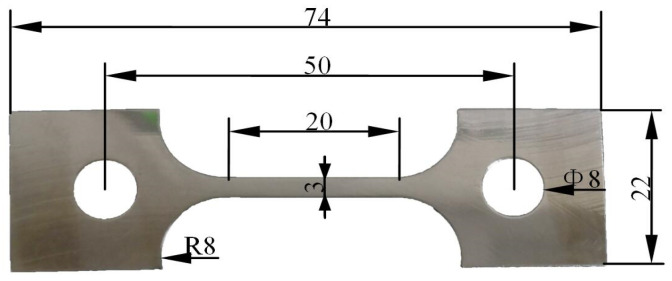
The plate tensile specimen geometric dimensioning (unit: mm).

In accordance with GB/T 228.1-2010, metallic materials are subjected to tensile testing at room temperature [25]. By taking into consideration the type of testing machine fixtures, a stainless steel sheet is meticulously crafted into a sheet-shaped tensile specimen, as illustrated in Figure 1, through the process of wire cutting. The gauge section of the specimen exhibits a section size of 3 mm × 2 mm and a length of 20 mm, following corresponding test standards of GB/T 2039-1997 [26]. Furthermore, the chamfer radius between the clamping section and the gauge section is precisely measured at 8 mm. To ensure that the surface roughness of the specimen remains below 3.2 μm, the surface is polished until a distinct metallic luster becomes apparent.

### 2.2. Utilizing Strain Gauges to Acquire Strain Data During Material Stretching

Resistance strain gauges are strain measurement components that operate based on the strain effect. The fundamental principle involves the mechanical deformation of strain gauges fixed on a structure when subjected to external loads, leading to changes in their resistance values. Through appropriate calculations, the strain variations in the structure can be determined. The specific experimental procedures for measuring the stress–strain curve of materials using a uniaxial tensile testing machine in conjunction with strain gauges involve the following steps:

(1) Strain gauge patch

Prior to pasting, the gauge section of the specimen is subjected to crosswise polishing along a 45° direction using fine sandpaper to enhance the bonding strength. Following polishing, the surface of the specimen is meticulously scrubbed with cotton yarn. In this particular test, acrylic epoxy resin adhesive is chosen as the adhesive material, and strain gauges are carefully affixed to the surface of the gauge section. The pertinent parameters of the resistance strain gauge BX120-3AA (Telesky, Shenzhen, China) employed in this study are comprehensively presented in Table 1. The parameters in Table 1 are based on the manufacturer’s specifications for the strain gauges used in this study.

(2) Connect strain gauge to deformeter

To facilitate the collection of strain data, the wires of the strain gauges are connected to the extension lines of the strain gauges through welding. This is achieved by utilizing a DH3818Y static strain tester (DONGHUA, Taizhou, China), which allows for the precise and accurate measurement of strain.

(3) Experiment process

The stretching rate of the tensile test is set to 2 mm/min. Following the configuration of the pertinent parameters within the strain acquisition software, both uniaxial tensile testing and strain data acquisition are executed simultaneously. Upon fracture of the specimen, the experiment is terminated. This experiment is carried out at ambient temperature conditions.

### 2.3. Utilizing DIC to Acquire Strain Data During Material Stretching

The fundamental principle of digital image correlation (DIC) involves the use of artificial speckle fields that are randomly or pseudo-randomly distributed on the surface of a test piece as carriers of deformation information. This enables the experimental mechanics method of full-field displacement and strain analysis of the surface of materials or structures under external load. During DIC testing, a three-dimensional adjustment bracket is utilized to adjust the position of speckle capture and control the leveling of the CCD camera. The light source is primarily used to regulate the intensity and convergence of light on the surface of the sample, resulting in a uniform light field. The recorded images of speckle variations on the sample’s surface are then analyzed to extract valuable information about the material’s mechanical behavior. The specific steps for measuring strain changes during the uniaxial tensile test of 316L stainless steel using DIC are as follows:

(1) Speckle spraying

**Figure 2:**
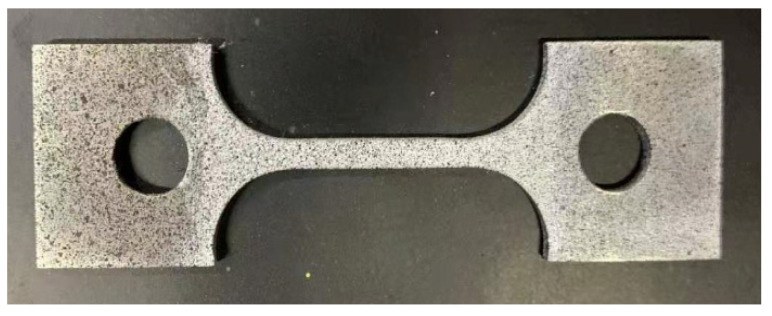
The plate tensile specimen following speckle spray treatment.

In this study, 316L stainless steel specimens of the same batch and size as those used in uniaxial tensile tests and strain gauge measurements were employed. The surface of each specimen was cleaned before being spray-painted to create a uniform speckle pattern with controlled paint concentration, as shown in Figure 2.

**Figure 3:**
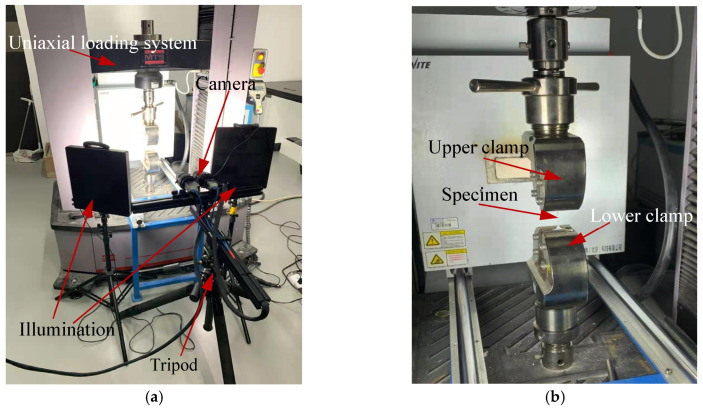
Experiment set-up of the tensile testing machine with DIC system. (**a**) DIC system composition. (**b**) The installation position of the specimen.

After speckle spray treatment, the specimens were vertically clamped onto the fixtures of the loading device. As shown in Figure 3a, the digital image correlation (DIC) measurement system used in this study is a VIC-2D system, which consists of a CCD camera, a light source, and a three-dimensional adjustment stand with a leveler. The data acquisition software used was VIC-Snap 8. An MTS-LPS.105 testing machine (MTS Systems Corporation, Eden Prairie, MN, USA) was employed as the material loading device, with a maximum load capacity of 100 kN. The clamping positions of the specimens are illustrated in Figure 3b.

(2) DIC device parameter adjustment

A CCD camera was mounted on a three-dimensional adjustment stand and aligned parallel to the ground using a leveler. The distances between the CCD camera, the light source, and the specimen were determined to ensure that the deformation of the gauge section of the specimen could be captured by the acquisition system. The light source was turned on to create a uniform light field on the surface of the specimen. The acquisition software was then run, and the aperture was adjusted to an appropriate position on the computer screen before adjusting the focal length until a clear and well-lit image appeared on the acquisition software interface.

(3) DIC software acquisition parameters and tensile speed settings.

The acquisition software (GOM Correlate, version 2020) was set to capture images at a frame rate of two frames per second. Prior to the start of the test, a photograph was taken at the unloaded state to establish a reference position for later software strain calculations. After setting the reference position, the uniaxial tensile test and image acquisition by the CCD camera were simultaneously initiated with a tensile speed of 2 mm/min using the testing machine.

## 3. Numerical Modeling

**Figure 4:**
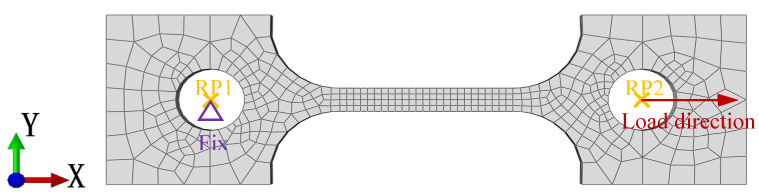
Mesh and boundary condition used for plate tensile specimen.

In this study, the ABAQUS software (version 2020) was used to complete the finite element analysis process. The FE model is shown in Figure 4, based on the dimensions of the tensile test specimen in Figure 1. For the boundary conditions, we set two reference points on the geometric model of the specimen. The left reference point (RP1) is fixed, and a displacement loading in the X-direction is applied to the center of the right reference point (RP2). The reference points (RP1 and RP2) couple with the inner surface of the loading hole of the specimen and the degrees of freedom outside the X direction are constrained to avoid possible rigid body motion. The code provided element C3D8 (8-node linear brick) was chosen. A total of 1176 elements were found with good convergence to catch the strain distribution on the gauge length of the specimen.

## 4. Results and Discussions

### 4.1. Strain Gauge Test Results

The engineering stress–strain curve (*σ*_eng_ − *ɛ*_eng_) of a material can be obtained through uniaxial tensile testing, and then the plastic parameters of the material can be obtained. It should be noted that the true strain (*ɛ*_true_) of the material is measured by the strain gauge, so it is necessary to convert the engineering stress (*σ*_eng_) obtained from the tensile machine to obtain the true stress–strain curve (*σ*_true_ − *ɛ*_true_) of the material. The conversion relationship between the engineering stress–strain curve and the true stress–strain curve (*σ*_true_ − *ɛ*_true_) is as follows:

(1) $$\sigma_{true}=\sigma_{eng}\left(1+\varepsilon_{eng}\right)$$

(2) $$\varepsilon_{true}=ln\left(1+\varepsilon_{eng}\right)$$

**Figure 5:**
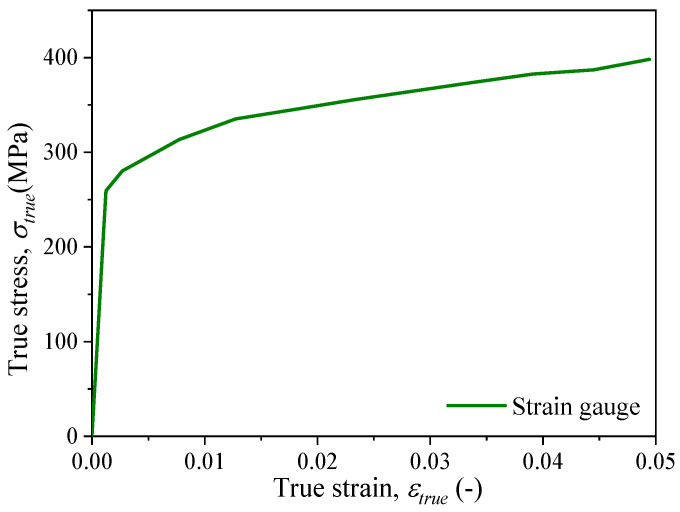
The true stress–strain curve from strain gauge.

The true stress–strain curve of the material, as measured, is depicted in Figure 5, where the green curve represents the true stress–strain curve of 316L stainless steel obtained through a strain gauge. It can be observed that due to the range limitation caused by the measuring principle of the strain gauge, the strain gauge fails when the strain value in the gauge section reaches approximately 0.05 during the testing process, causing a discontinuity in the curve and rendering it impossible to continue measuring the mechanical property curve of the material. During numerical simulations, significant deformation occurs at locations of stress concentration. To ensure the reliability of the numerical simulation results, a strain data measurement method with a wider range needs to be employed during testing. Notably, all experimental groups demonstrated mechanical response consistency within 2% relative standard deviation, with graphical presentation optimized through the selection of representative datasets from strain gauge and DIC measurement systems.

### 4.2. DIC Results Comparison

**Figure 6:**
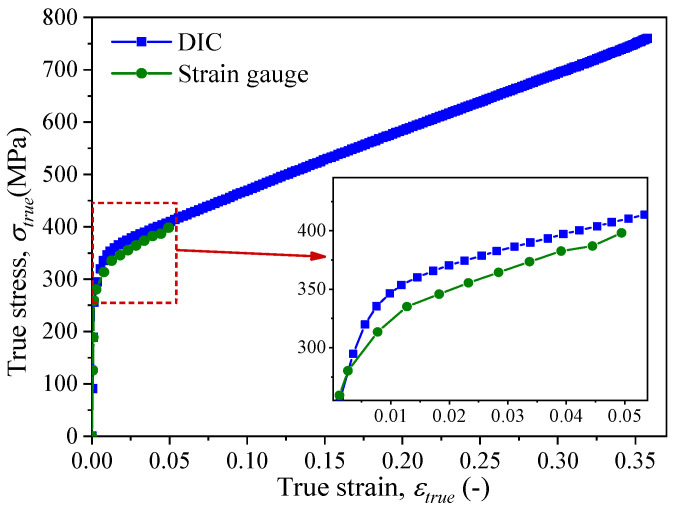
Comparison of the true stress–strain curve from DIC and stain gauge.

To obtain the corresponding true stress–strain curve, the engineering stress (*σ*_eng_) from the tensile testing machine was converted to true stress (*σ*_true_) using Equations (1) and (2). Subsequently, a comparison between the stress–strain curves obtained by the resistance strain gauge and DIC method is shown in Figure 6. A magnified inset was incorporated to highlight the measurement domain of the strain gauge, facilitating the precise observation of strain localization characteristics within the elastic deformation regime. The results demonstrate that within the measurement range of the resistance strain gauge, both methods yield consistent true stress–strain curves for the material, thereby validating the reliability of the DIC method. Minor discrepancies observed in the initial stage may originate from specimen manufacturing tolerances and slight variations in strain gauge bonding accuracy.

The comparison between the two methods reveals that the measurement range of resistive strain gauges is relatively limited, and they cannot function properly when the strain exceeds approximately 0.05. Consequently, resistive strain gauges are unable to obtain full-field strain data from the tested sample. In contrast, non-contact optical measurement methods, such as DIC, can reliably output full-field strain data from the tested sample with a measurement range that extends up to the point of fracture, which is significantly higher than that of strain gauges. Therefore, during numerical simulations, it is feasible to select the true stress–strain data obtained through DIC as the material mechanical property parameters in the finite element model.

**Figure 7:**
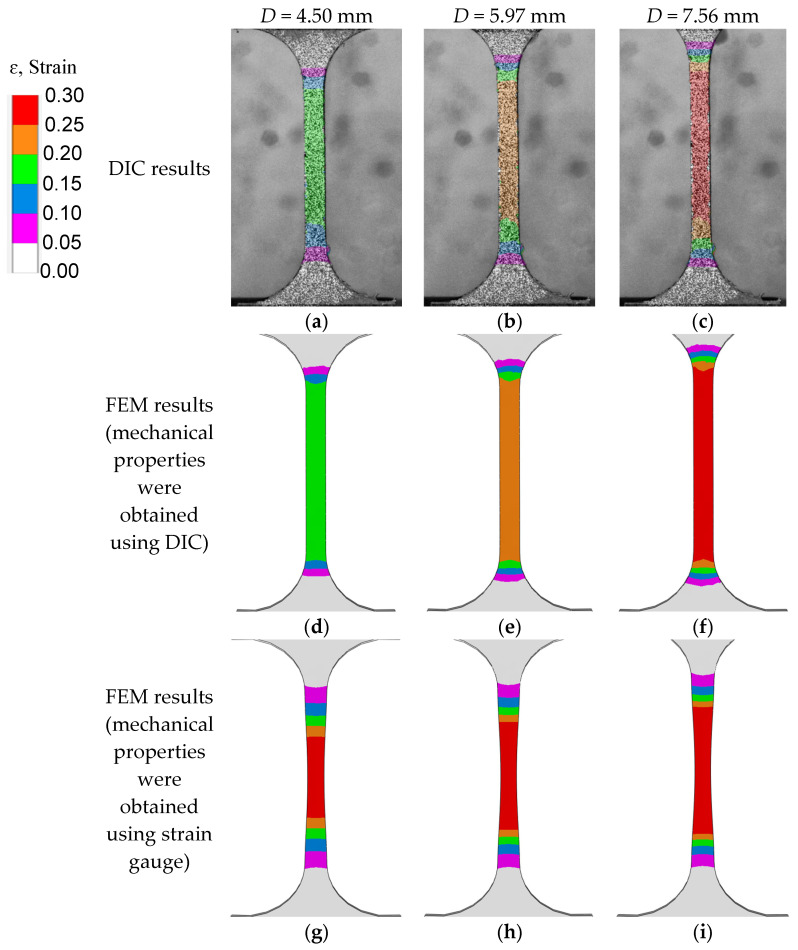
The changes in strain with different mechanical parameters.(**a**) D = 4.50 mm (DIC result); (**b**) D = 5.97 mm (DIC result); (**c**) D = 7.56 mm (DIC result); (**d**) D = 4.50 mm (FFM result using DIC data); (**e**) D = 5.97 mm (FFM result using DIC data); (**f**) D = 7.56 mm (FFM result using DIC data); (**g**) D = 4.50 mm (FFM result using strain gauge data); (**h**) D = 5.97 mm (FFM result using strain gauge data); (**i**) D = 7.56 mm (FFM result using strain gauge data).

The strain cloud diagram of the gauge section of the sample during the tensile process can be obtained through processing using GOM Correlate software, as shown in Figure 7a–c. Since the lower clamp of the tensile testing machine remains fixed, the load is applied through displacement of the upper clamp. Therefore, the symbol *D* in Figure 7 represents the displacement of the specimen at the gripping location of the upper clamp. From the figures, it can be observed that with the continuous increase in load, the strain value of the gauge section also increases. During the uniaxial tensile test, the strain distribution of the gauge section is relatively uniform, and data on the variation in strain values of the gauge section with time can be outputted through the software. The computational results of the finite element models with different material parameters are shown in Figure 7d–i. Among them, Figure 7d–f shows the strain distribution obtained through finite element calculation using DIC test results as the material mechanical property parameters, while Figure 7g–i uses the strain gauge test results as the material mechanical property parameters. From the figures, it can be observed that when the material undergoes significant plastic deformation, the computational results in Figure 7d–f are generally consistent with the experimental (DIC) results. However, due to limited data based on strain gauge measurements, the finite element computation results deviate from the experimental results at higher levels of plastic deformation. The DIC technique enables full-field strain measurements (as demonstrated in Figure 6), capable of resolving strain distributions in regions inaccessible to conventional strain gauges (e.g., high-strain gradient zones), though its implementation is hindered by the high cost of speckle-recording equipment, limiting accessibility in budget-constrained settings.

The accuracy of DIC-measured strains aligns with recent studies on nuclear materials. For example, Zhang et al. demonstrated that DIC achieves ±0.1% strain accuracy for austenitic steels under quasi-static loading, consistent with our findings [16]. During necking, our observed strain localization matches the patterns in 316L steel reported by Jordan et al., who combined DIC with finite element modeling [27].

## 5. Conclusions

The following conclusions can be drawn from this study:

(1) When the sample under study experiences significant deformation, the limited measurement range of the strain gauge can impose constraints on the quantity of strain data that can be collected. This, in turn, has a substantial impact on the acquisition of accurate mechanical property parameters. The restricted gauge range acts as a bottleneck, hindering the comprehensive characterization of the material’s mechanical behavior during large-scale deformation processes.
(2) The utilization of the DIC technique for strain measurement offers a distinct advantage in enabling precise strain testing over a significantly broader range. This broader measurement scope allows for the collection of more comprehensive material mechanical parameters, which are crucial for conducting accurate elastoplastic finite element simulations. The DIC method provides a more holistic view of the material’s strain evolution, thereby enhancing the reliability and accuracy of the finite element models.
(3) On the premise of accurately determining the mechanical property parameters of the material, the finite element simulation-derived strain distribution of 316L steel has been found to be in excellent agreement with the strain distribution obtained through DIC measurements. This congruence validates the effectiveness of the DIC method as an essential validation tool for finite element simulations. The DIC technique not only provides reliable experimental data but also serves as a benchmark for validating the numerical results of finite element models, thereby contributing to the overall improvement of the simulation accuracy and the understanding of the material’s mechanical behavior.

For the purpose of structural integrity analyses of key nuclear structures such as reactor pressure vessels (RPVs) and primary circuit pipelines, many scholars and research institutions are making efforts to predict the mechanical properties after long-term service. The validated accuracy of DIC in characterizing homogeneous 316L stainless steel strongly supports its potential application in quantifying the mechanical behavior of heterogeneous nuclear structures, such as welded joints or defect-containing components—critical yet understudied scenarios in primary circuit pipelines. Building on our methodology, where DIC achieved relatively high strain resolution on polished specimens, the technique could map strain localization near weld seams or crack tips in irradiated/welded 316L, overcoming the spatial limitations of strain gauges. For instance, our speckle preparation protocol could be adapted to create controlled artificial defects (e.g., micro-notches mimicking stress corrosion cracks) to study strain redistribution during damage progression. The FE framework, currently calibrated using homogeneous material data, could integrate DIC-derived heterogeneous strain fields to refine constitutive models for weld zones, where microstructure variations (e.g., heat-affected zone and base metal) induce mechanical property gradients. Further study has been planned along this line.

## Figures and Tables

**Table 1:** Parameters of strain gauge.

Substrate Material	Wire Material	Length and Width (mm)	Resistance Ω	Sensitivity Coefficient	Nominal Voltage
acetal	constantan	6.5 × 3.5	120.0 ± 0.3	2.06 ± 0.01	≤12 V

## Data Availability

The original contributions presented in this study are included in the article. Further inquiries can be directed to the corresponding author.
